# Autoimmune pancreatitis with colonic stenosis: an unusual complication and atypical pancreatographic finding

**DOI:** 10.1186/1471-230X-14-173

**Published:** 2014-10-03

**Authors:** Hiroyuki Matsubayashi, Yoshihiro Kishida, Yukio Yoshida, Masao Yoshida, Yasuyuki Tanaka, Kimihiro Igarashi, Kenichiro Imai, Hiroyuki Ono

**Affiliations:** Division of Endoscopy, Shizuoka Cancer Center, Nagaizumi, Suntogun, Shizuoka, 411-8777 Japan

**Keywords:** Autoimmune pancreatitis, Colonic stenosis, Differential diagnosis, Steroid therapy

## Abstract

**Background:**

Type 1 autoimmune pancreatitis (AIP) often accompanies various systematic disorders such as sclerosing cholangitis, sialoadenitis, retroperitoneal fibrosis, interstitial pneumonitis and nephritis. Although rarely reported in acute pancreatitis, colonic stenosis is an uncommon complication in cases with AIP.

**Case presentation:**

A 69-year-old Japanese man complained of abdominal pain and continuous diarrhea, resistant to intake of antimuscarinic and probiotic agents. A colonoscopy demonstrated a stenosis at the splenic flexure. Computed tomography revealed a focal enlargement of the pancreatic tail with a capsule-like rim, contacting with the descending colon. Endoscopic retrograde pancreatography (ERP) was unable to visualize the main pancreatic duct (MPD) at the pancreatic tail, despite a full contrast injection. A high serum IgG4 level (1060 mg/dL) and exclusion of pancreatic cancer by endoscopic ultrasound guided-fine needle aspiration suggested AIP, but did not fulfill the diagnostic criteria, and steroid therapy was initiated. One month after starting steroid intake, pancreatic swelling was minimized and the MPD was visualized by ERP, fulfilling the international consensus diagnostic criteria (ICDC) of AIP. Colonic stenosis was relieved and the patient’s symptoms disappeared.

**Conclusion:**

The present case is the first report of AIP developing colonic stenosis by the inflammatory infiltration. In this case, steroid therapy was effective for the diagnosis and treatment of pancreatic mass involving the descending colon.

## Background

Cases of autoimmune pancreatitis (AIP) mostly manifest as complaints of abdominal pain, jaundice, dullness, weight loss, development or worsening of diabetes mellitus, and symptoms associated with other organ involvements (OOI). Type1 AIP, characterized by narrowing of the main pancreatic duct (MPD) by lymph plasmacytic sclerosing pancreatitis and high levels of serum IgG4, often accompanies OOI such as the lachrymal and salivary glands, lung, biliary tract, liver, kidney, retroperitoneum, urinary tract, and prostate [1, 2]. This report presents a case of AIP accompanied by stenosis of the MPD and of the descending colon.

## Case presentation

**Figure 1:**
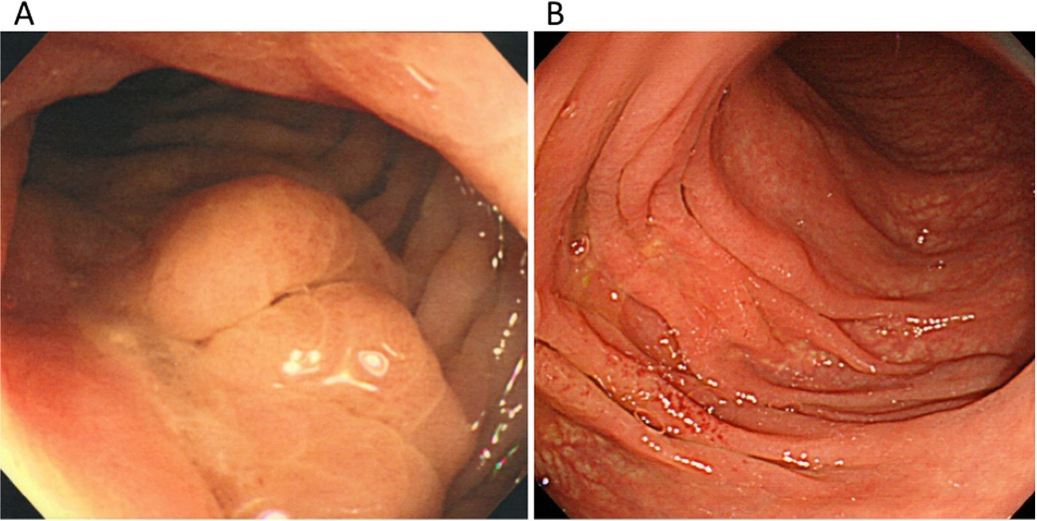
**Colonoscopic views at the splenic flexure before (A) and after (B) steroid therapy.** The erosion associated with a converging fold and luminal stenosis **(A)**. Healed erosion with an indistinct mucosal vascular pattern **(B)**.

**Figure 2:**
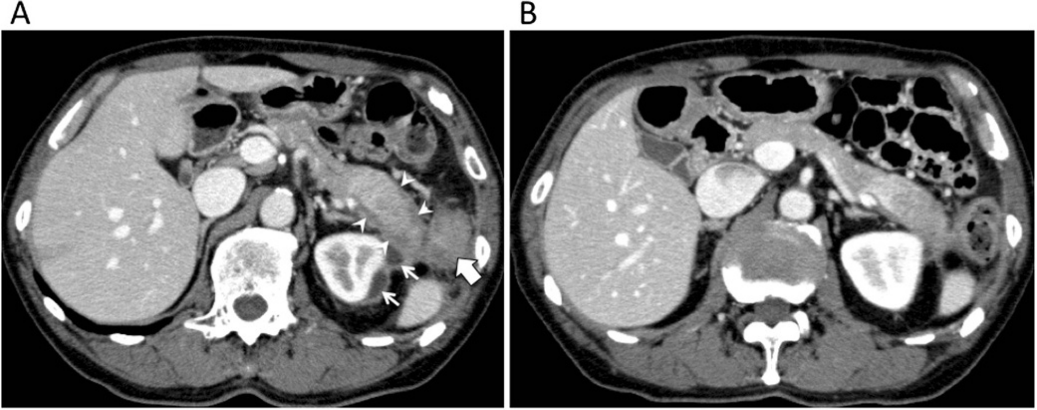
**Enhanced computed tomography showing an enlarged pancreatic tail, before (A) and after (B) steroid initiation.** A swollen pancreas, adhesive to the descending colon (large arrow), with a capsule-like rim (arrow head) and effusion (small arrow) around the left kidney **(A)**. Minimized pancreatic swelling and decreased effusion **(B)**.

**Figure 3:**
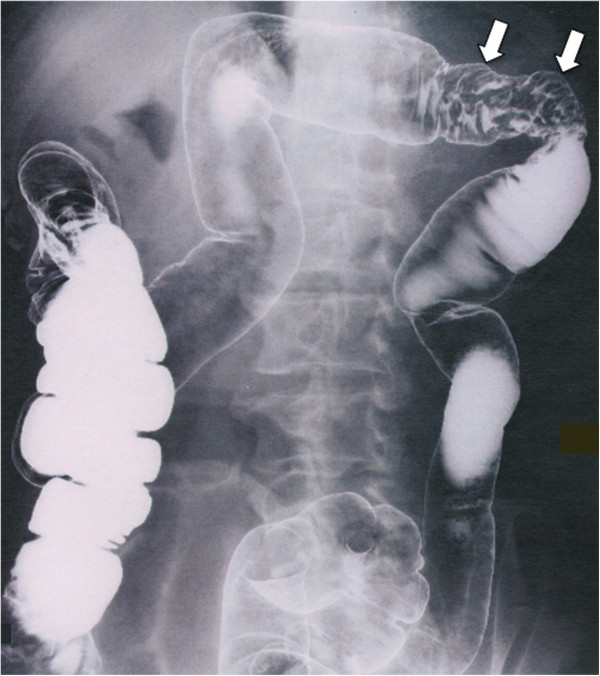
**Contrast enema showing an irregular stenosis of the colon at the splenic flexure.**

**Figure 4:**
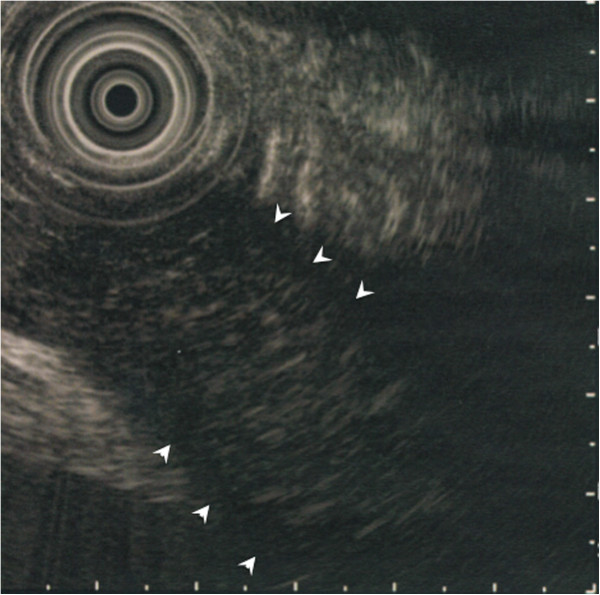
**Endoscopic ultrasonographic view showing the low-echoic, enlarged pancreatic tail with a marginal capsule-like rim (arrowhead).**

**Figure 5:**
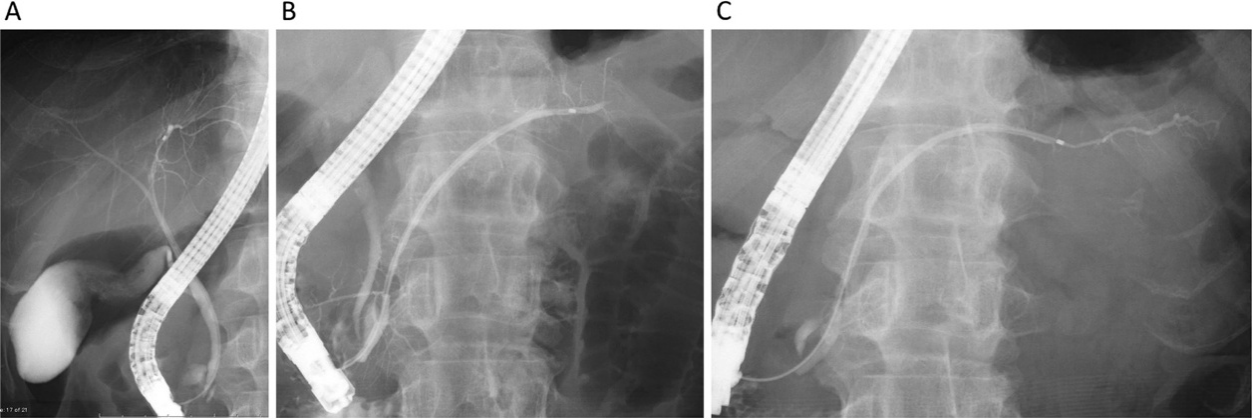
**Endoscopic retrograde cholangiopancreatography.** Thin structure of the intrahepatic bile duct **(A)**. Pancreatography showing a stenosis of the main pancreatic duct before steroid therapy **(B)**. Reopening of the main pancreatic duct after the steroid initiation **(C)**.

**Figure 6:**
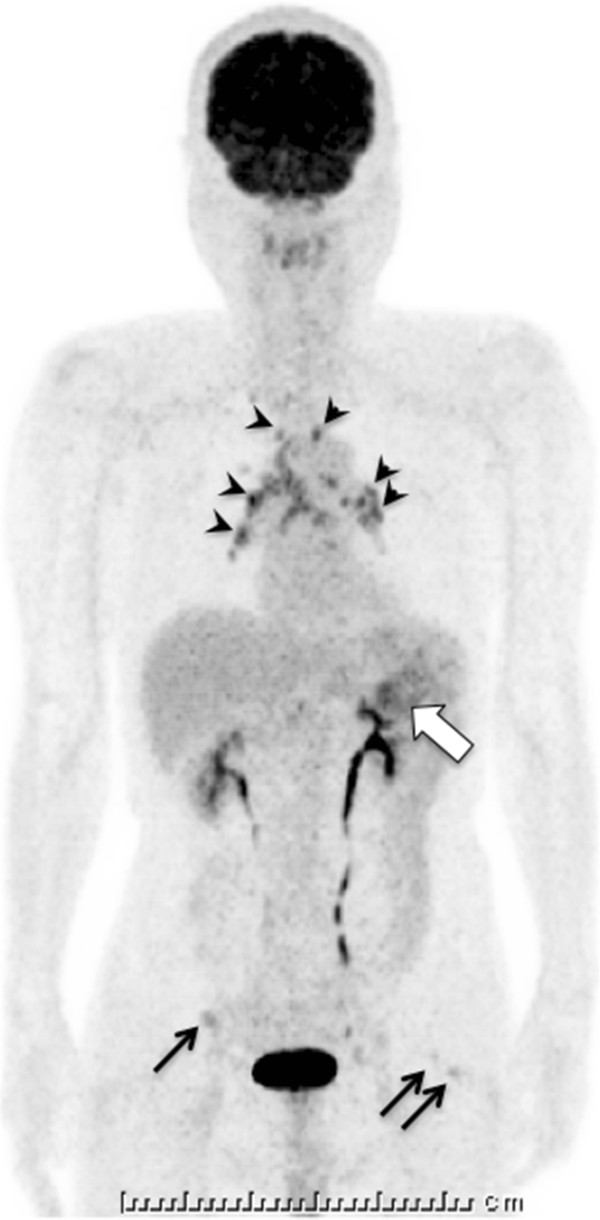
^**18**^ **F-fluorodeoxyglucose positron emission tomography showing abnormal uptake of** ^**18**^ **F-fluorodeoxyglucose at the pancreatic tail (large arrow), as well as at the lymph nodes of the mediastinum (arrowhead) and inguinalis (small arrow).**

A 69-year-old Japanese man suffered from intermittent abdominal pain and repetitive diarrhea for a month, and visited the nearest hospital. He had habits of drinking alcohol and smoking. His past history included only prostate hypertrophy and his family history was unremarkable. A colonoscopy (Figure 1A) revealed mucosal erosion accompanied with converging folds and luminal stenosis at the splenic flexure. Biopsy tissue obtained from the erosion showed the histology of non-specific colitis without neoplastic cells [IgG4-positive cells: 2 cells/high power field (HPF)]. Computed tomography (CT) (Figure 2A) revealed a focally enlarged pancreatic tail with heterogeneous contrast enhancement. The tail of the pancreas showed soft tissue proliferation surrounding the pancreas that appeared to adhere to the descending colon, accompanied with small amount of effusion. A contrast enema (Figure 3) revealed an irregularly narrowed segment of the large bowel at the splenic flexure. His symptoms had been resistant to any medications (e.g., tiquizium bromide, camostat mesilate, or miyarisan) and he had lost 7 kg of weight during the previous two months. Pancreatic tail cancer invading to the colon had to be ruled out, so the patient was referred to our hospital. Blood tests showed an increasing level of serum amylase (703 U/L) and c reactive protein (3.0 mg/dL). Serum tumor markers (carcinoembryonic antigen and carbohydrate antigen 19–9) and HbA1c were within the normal range, but serum IgG (2661 mg/dL: ≤1700 mg/dL), IgG4 (1060 mg/dL, normal: ≤105 mg/dL), and IgE (317 IU/mL, normal: ≤173 IU/mL) were all elevated. Antinuclear antibody and rheumatoid arthritis particle agglutination were also increased in the serum (positive at ×80 and ×160 dilution, respectively). Endoscopic ultrasound (EUS) showed mosaic high echoic foci within the low-echoic, swollen pancreatic tail, with a capsule-like rim structure (Figure 4). Endoscopic retrograde cholangiography (ERC) showed diffusely thin intrahepatic bile ducts (Figure 5A). Pancreatography demonstrated a stenosis of the MPD despite the high pressure of contrast injection (Figure 5B) [3]. Intraductal ultrasound of the bile duct showed no remarkable findings [4]. Biopsied tissues from the bile duct and major papilla were non-neoplastic and showed only a scattering of IgG4-positive cells (3 cells/HPF). On the following day, the patient complained of mild left hypochondralgia and had a high level of serum amylase (667 U/L), suggesting a development of mild post-ERCP pancreatitis. Pancreatic cancer was excluded by an EUS-guided fine needle aspiration biopsy (FNAB) [5] performed from the gastric body using a 22-gauge needle (Echotip®, COOK, Bloomington, USA). The obtained specimens showed neither cancer tissue nor IgG4-positive plasma cells. ^18^ F-fluorodeoxyglucose positron emission tomography (FDG-PET) demonstrated abnormal uptakes at the pancreatic tail (SUVmax: 7.2), and at the lymph nodes of mediastinal and inguinal region (Figure 6). No OOIs suggestive of AIP, such as biliary stricture, retroperitoneal fibrosis, enlargement of salivary or lachrymal glands, were apparent in any of the image examinations. A rapid steroid trial was initiated with 30 mg/day of prednisolone based on the suspected diagnosis of AIP and the continued need to exclude pancreatic cancer, since the imaging findings did not fulfill any of the diagnostic criteria for AIP [1]. A steroid response was not obvious at two weeks after steroid initiation, but improvement in the pancreatic tail enlargement and peripancreatic effusion was recognized by abdominal ultrasonography (US) and CT at one month after steroid initiation (Figure 2B). The second look ERCP demonstrated a non-remarkable change in the biliary tract but obvious improvement of stenosis of the MPD (Figure 5C). The following CF (Figure 1B) confirmed the wide opening of the colon. This steroid response satisfied the international consensus diagnostic criteria (ICDC) [2], but did not fulfill the Japanese criteria (2011) [1]. The patient’s symptoms disappeared and his subsequent progress was uneventful.

## Discussion

The current case showed an increasing level of serum IgG4 and a colonic stenosis without evidence of inflammatory bowel disease; hence, it is thought to be a type1 rather than a type2 AIP. Colonic manifestation has seldom been reported as a complication in cases of type1 AIP [1, 2]. The initial ERP demonstrated a stenosis of the MPD, but not a narrowing [3]. These findings confounded the diagnosis of current case as AIP, and corticosteroid administration played important roles both in diagnosis and in therapeutic aspects.

The diagnostic criteria of AIP define the pancreatographic finding as a long or segmental “narrowing” of the MPD [1–3], but not as a stenosis or obstruction. The finding upstream of the stricture that is described in the ICDC [1, 2] can only be visualized by high pressure injection of the contrast, but this makes post-ERCP pancreatitis a concern, especially in cases with a pancreatic tail lesion. Actually, in the current case, the initial pancreatography demonstrated a stenosis of the MPD even with a considerably high pressure of contrast injection; thus, pancreatography may worsen the pancreatitis. Hence, current case did not meet the diagnostic criteria of ICDC before steroid therapy [1, 2].

The lack of definitive histological evidence and typical OOIs meant that this case did not meet the any of diagnostic criteria of AIP, until the steroid trial. Usually a steroid response should be determined over a short interval [1] and we anticipated a response visible by US but failed to confirm this within two weeks. Our experience using abdominal US is that an obvious downsizing of pancreatic lesion is seen in 86% of AIP cases within two weeks of steroid administration [6]. However, evaluation of the margin of the pancreatic tail lesion with surrounding effusion may be difficult using US. In these atypical cases, confirming the reopening of the stenotic MPD or reexamination by EUS-FNAB is likely to be necessary, as in current case. The steroid response ultimately seen in our case meant that it met the criteria of the ICDC [2], but not Japanese criteria (2011), which requires “irregular narrowing of the MPD” in any set of diagnostic criteria when a definitive pathological diagnosis is lacking [1]. Future revision of the criteria should take the present findings into consideration.

Colonic involvement has been very infrequently reported in a small number of cases with conventional acute pancreatitis (<50 cases listed by PubMed keyword survey), and mostly at the splenic flexure as in the present case. These occurrences were confused with a carcinoma based on endoscopic, radiologic, or laparotomic findings [7]. A colonic stenosis is thought to arise either as a consequence of ischemia and necrosis due to active pancreatitis or because of compression due to a pancreatic pseudocyst [8, 9]. Colonic obstruction arising during the acute phase of pancreatitis usually is spontaneously reversible, but operation is required in cases with colonic necrosis or persistent intestinal obstruction. Cases with severe necrosis sometimes develop adjacent abscesses, perforations, or peritonitis [7] and cannot be rescued even with surgery (6 out of 22 surgical cases, reported by Aldridge et al. in 1989) [10]. In our case, according to the clinical images (Figures 1, 2 and 3) and biopsy obtained from the colonic erosion (low level of IgG4-positive cells), the colonic stricture was supposed to be secondary to the inflammation caused by AIP, rather than the colonic manifestation of IgG4-related systemic disorders. The level of the stricture fortunately was not severe, but the colonic stenosis associated with adjacent effusion and the continuous nature of the patient’s symptoms necessitated intrinsic treatments. In this patient, a pancreatic enzyme inhibitor and intestinal regulators were not effective but the corticosteroid worked well.

## Conclusion

The current case with AIP was highly suggestive from the viewpoints of colonic involvement and the lack of visualization of the MPD. The steroid trial was effective in both diagnosis and therapy in this case.

## Consent

Written informed consent was obtained from the patient for publication of this case report and any accompanying images. A copy of the written consent is available for review by the editor of this journal.

### IRB approval

Institutional Review Board of Shizuoka Cancer Center ethically approved this case report (Institutional code number: 25-J122-25-1-3).

## Abbreviations

AIP: Autoimmune pancreatitis
ERP: Endoscopic retrograde pancreatography
MPD: Main pancreatic duct
ICDC: International consensus diagnostic criteria
OOI: Other organ involvements
HPF: High power field
CT: Computed tomography
EUS: Endoscopic ultrasound
ERC: Endoscopic retrograde cholangiography
FNAB: Fine needle aspiration biopsy
FDG-PET: ^18^ F-fluorodeoxyglucose positron emission tomography
US: Ultrasonography.
